# On Accounting for Sequence-Specific Bias in Genome-Wide Chromatin Accessibility Experiments: Recent Advances and Contradictions

**DOI:** 10.3389/fbioe.2015.00144

**Published:** 2015-09-22

**Authors:** Pedro Madrigal

**Affiliations:** ^1^Wellcome Trust Sanger Institute, Cambridge, UK; ^2^Department of Surgery, University of Cambridge, Cambridge, UK

**Keywords:** next-generation sequencing, DNase-seq, ATAC-seq, chromatin accessibility, footprinting, sequence bias, ChIP-exo

## Next-Generation Sequencing for Chromatin Biology

Uncovering the protein–DNA interactions involved in cell fate, development, and disease in a time- and cell-specific manner is a fundamental goal of molecular biology. The advent of the sequencing technologies has opened a new genomic era, uncovering the information encoded in genomes, epigenomes, and transcriptomes (McPherson, 2014). For example, the popular ChIP-based techniques ChIP-seq (Johnson et al., 2007; Robertson et al., 2007) and ChIP-exo (Rhee and Pugh, 2011) are widely used to detect transcription factor (TF)-binding sites using an antibody against a single protein of interest (Mahony and Pugh, 2015). Alternative protocols assaying the chromatin landscape, such as those based on digestion by DNase I enzyme (DNase-seq), micrococcal nuclease (MNase-seq), and Tn5 transposase attack (ATAC-seq), enable the identification of DNA-binding protein footprints of many TFs in a single experiment (Tsompana and Buck, 2014). Time-series experiments might be required for the identification of those TFs cataloged as pioneer factors, allowing their effects on chromatin to be investigated (Zaret and Carroll, 2011; Pajoro et al., 2014; Sherwood et al., 2014).

Despite the initial promise of detecting the majority of TFs in one assay, DNA sequence-specific biases, together with TF-dependent binding kinetics, have been recently pinpointed as major confounding factors in DNase-seq experiments (Koohy et al., 2013; He et al., 2014; Raj and McVicker, 2014; Rusk, 2014; Sung et al., 2014). These influencing factors were not considered by any of the previous computational approaches for the analysis of next-generation sequencing chromatin accessibility data (Madrigal and Krajewski, 2012); neither those strategies based on TF-generic DNase signature nor those based on TF-specific DNase signature (Luo and Hartemink, 2013).

## Alleviating Sequence-Specific Biases in DNase-seq

To partly address these challenges, four recent approaches have been published that model, predict, or explain DNase I sequence specificity in order to improve the detection of TF occupancy events at high resolution (digital genomic footprinting). The first method, FootprintMixture, uses a multinomial mixture model in which one mixture models the footprint component, and the other the background component taking into account the sequence bias (Yardimci et al., 2014). The background can be either uniform or derived from naked DNA measurements – this is the main difference with respect to the footprint component in CENTIPEDE (Pique-Regi et al., 2011), which assumes a uniform background. Alternatively, more than two components may be set to detect variability in the footprint model. Thus, the cleavage signature (number of DNase I cuts that map to each nucleotide) is used in a multinomial mixture model to classify candidate sites as either “bound” or “unbound” aided by 6-mer DNase sequence bias cleavage frequencies (Yardimci et al., 2014). Remarkably, the authors found that sequence bias is DNase-seq protocol specific. They also found that the signature of a footprint could be formed by a mixture of DNase digestion profiles identified by unsupervised *k*-means clustering, in agreement with the observations found in an earlier study (Tewari et al., 2012). For TFs CTCF and ZNF143, variants of the consensus sequence motif associated to different footprint shapes were observed.

In the second, the DNase2TF algorithm is able to correct dinucleotide bias, detecting footprints with accuracy better or comparable to existing approaches (Sung et al., 2014). Furthermore, Sung et al. (2014) were able to predict DNase signatures using solely tetranucleotide frequency information. Although this 4-nucleotide region has the highest information content, Koohy et al. (2013) and Lazarovici et al. (2013) demonstrated information beyond a context longer than four nucleotides. Consequently, using naked (deproteinized) DNA control datasets specific to a protocol and an enzyme as well as high sequencing depth (Hesselberth et al., 2009) are now suggested recommendations for DNase-seq experiments aiming to detect footprints (Meyer and Liu, 2014).

A third approach, an improved version of HINT [HMM-based identification of TF footprints (Gusmao et al., 2014)], named as HINT-BC/HINT-BCN (Bias Correction based on hypersensitivity sites/Bias Correction based on Naked DNase-seq) includes *k*-mer based bias correction in DNase-seq data as in He et al. (2014), leading to substantial changes in the average DNase I cleavage patterns surrounding the TFs. These changes result beneficial to footprinting method accuracy (personal communication with the author).

Contradictorily, a fourth study using DNase-seq has shown that bias correction does not significantly improve the accuracy of TF binding identification (Kähärä and Lähdesmäki, 2015). In addition, this study poses a second counterintuitive idea in the field: accuracy saturates at a modest sequencing depth (30–60 million reads), and only a few TFs present improvement at deeper sequencing.

## ATAC-seq Shows Sequence Cleavage Bias

**Figure 1:**
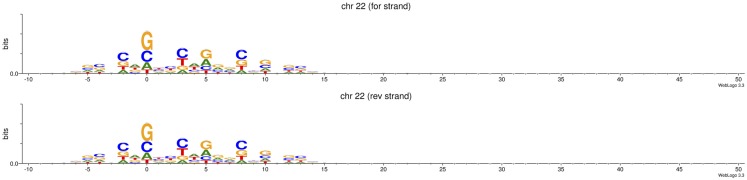
**Tn5 transposase shows sequence cleavage bias**. Data represented correspond to read-start sites in reads aligned to forward and reverse strands in chromosome 22 in four ATAC-seq replicates (50 k cells per replicate) reported in Buenrostro et al. (2013). Of total, 50 bp PE reads were pre-processed with Trimmomatic v0.32 under default parameters, and then aligned to hg19 using BWA v0.7.4-r385 (Li and Durbin, 2010; Bolger et al., 2014). Sequence logos were generated using WebLogo (Crooks et al., 2004). *Y* -axis: 0.0–0.3 bits.

It is unknown if ATAC-seq derived footprints are factor dependent or affected by Tn5 cleavage preferences (Tsompana and Buck, 2014). As expected, bioinformatic analysis of chromosome 22 in the published human datasets for 50,000 cells reveals sequence biases in ATAC-seq experiments (Buenrostro et al., 2013) (Figure 1), similar to those found by Koohy et al. (2013) in DNase-seq. As ATAC-seq might replace DNase-seq in the foreseeable future due to its cost and time efficiencies, and because it simultaneously allows the identification of nucleosome positions (Buenrostro et al., 2013), new computational models are necessary to evaluate intrinsic confounding factors in ATAC-seq.

A novel approach, msCentipede (Raj et al., 2014), has extended CENTIPEDE (Pique-Regi et al., 2011) from a mutinomial model to a hierarchical multiscale model. It has been evaluated on “single-hit” UW DNase-seq (Hesselberth et al., 2009) and on paired-end (PE) ATAC-seq data. Surprisingly, the “flexible model” for background DNase I cleavage rate (msCentipede-flexbg) shows very little improvement for a broad range of factors when taking into account naked DNA information from Lazarovici et al. (2013) datasets. This finding clearly contradicts those of He et al. (2014) and Sung et al. (2014). In msCentipede, the footprint signature (or cleavage profile) pattern within a factor-bound motif instance was, therefore, found to be informative when increasing the sensitivity and specificity of the TF binding site prediction. Raj et al. (2014) suggest that this might be explained by the different range of read count data between the matched consensus sequence of the candidate site/motif (10–30 bp) and the data matrix used typically by the software packages (larger sequence window, around 100–150 bp extension at each flank of the motif), which can mask the effects produced by not accounting for sequence biases within the core motif.

## Are Current Benchmarks Adequate to Evaluate Bias-Corrected DNase-seq Data?

So far, a footprint of a TF, therefore, might be either detectable (and better detectable when accounting, or not, for influencing factors), or undetectable. In many studies, both problems are convoluted and addressed using the same “gold standard” datasets, such as ChIP-seq, which do not have nucleotide-level resolution. Hence, on these methods and gold standards, no reproducible improvements can be seen. This was already noted in Cuellar-Partida et al. (2012), when it was showed that simply scanning for position weight matrices in DNase I hypersensitive sites (DHSs) had the same power as CENTIPEDE. These issues also complicate data integration with TF ChIP-seq, as peaks without a footprint in DNase-seq/ATAC-seq, considered weak/indirect binding or false positives (ChIP artifacts), might instead be explained by a class of TFs with rapid kinetics. And vice versa, DNase I cleavage patterns located within “ChIP-seq unbound” sites – noted previously, e.g., in the MILLIPEDE framework, especially in yeast (Luo and Hartemink, 2013) – could support the hypothesis of footprint shape dominated by DNA sequence specificities.

## Future Directions

There is room for improvement in current methodologies by making use of the sequence specificity of each enzyme/assay, including ATAC-seq, but there is no clear consensus in its importance for digital genomic footprinting. This situation is not exclusive for genome-wide chromatin accessibility experiments: modeling the sequence-specific lambda exonuclease bias in ChIP-exo did not significantly increase the identification of TF binding sites (Wang et al., 2014). Similarly, there is no clear consensus if footprint signatures at the core motif, whether they are unique or not for an individual factor, are really important for footprint identification. Establishing better benchmarks to compare performance of the algorithms across different protocols is a fundamental task. These benchmarks could be based on “differential footprints” (sites within DHSs that are bound by a factor in one condition but not the other) as a more appropriate metric to evaluate footprint identification performance instead of using ChIP-seq data (Yardimci et al., 2014). In addition, are DNase-seq software tools equally applicable to ATAC-seq without modification? If enzyme-specific biases are taken into account in a comparable experimental set-up, will DNase-seq and ATAC-seq report the same footprints for an identical sample using same algorithm parameters? This is unlikely, based on a previous comparison between open chromatin DHSs and FAIRE sites, which revealed unique regions produced in each assay (Song et al., 2011). It has been also proposed that performing, and combining, experiments with different nucleases can be an alternative to mitigate biases (He et al., 2014; Mahony and Pugh, 2015).

A greater challenge is dealing with proteins with very short residency time in the DNA as they produce mostly negligible footprints (Rusk, 2014; Sung et al., 2014). Optimizing and implementing new methods is necessary in order to enable biological insights that current methods cannot reveal.

## Conflict of Interest Statement

The author declares that the research was conducted in the absence of any commercial or financial relationships that could be construed as a potential conflict of interest.
